# Molecular Characterization of *Streptococcus pneumoniae* Strains Isolated from Patients with Invasive Infections in a Romanian Hospital

**DOI:** 10.3390/microorganisms14020418

**Published:** 2026-02-10

**Authors:** Endre Csaba Pál, Hunor Váradi, Attila Bitai, Mihaela Oprea, Sorin Dinu, Laura-Ioana Popa, Előd Ernő Nagy, Edit Székely

**Affiliations:** 1 Doctoral School of Medicine and Pharmacy, George Emil Palade University of Medicine, Pharmacy, Science, and Technology of Targu Mures, 38 Gheorghe Marinescu Street, 540142 Targu Mures, Romania; 2 Faculty of Medicine, George Emil Palade University of Medicine, Pharmacy, Science, and Technology of Targu Mures, 38 Gheorghe Marinescu Street, 540142 Targu Mures, Romania; varadi.hunor@yahoo.com (H.V.); bitai.attila.22@stud.umfst.ro (A.B.); 3 Laboratory of Molecular Epidemiology for Communicable Diseases, Cantacuzino National Medical-Military Institute for Research and Development, 103 Splaiul Independenței, 050096 Bucharest, Romania; oprea.mihaela@cantacuzino.ro (M.O.); dinu.sorin@cantacuzino.ro (S.D.); 4 Genomics, Transcriptomics, and Bioinformatics Platform, Cantacuzino National Medical-Military Institute for Research and Development, 103 Splaiul Independenței, 050096 Bucharest, Romania; popa.laura@cantacuzino.ro; 5 Department of Biochemistry and Environmental Chemistry, George Emil Palade University of Medicine, Pharmacy, Science, and Technology of Targu Mures, 38 Gheorghe Marinescu Street, 540142 Targu Mures, Romania; elod.nagy@umfst.ro; 6 Department of Microbiology, George Emil Palade University of Medicine, Pharmacy, Science, and Technology of Targu Mures, 38 Gheorghe Marinescu Street, 540142 Targu Mures, Romania; edit.szekely@umfst.ro; 7 Medical Microbiology Laboratory, County Emergency Clinical Hospital Targu Mures, 38 Gheorghe Marinescu Street, 540142 Targu Mures, Romania

**Keywords:** *Streptococcus pneumoniae*, invasive pneumococcal disease, pneumococcal serotype, pneumococcal conjugate vaccine, whole genome sequencing

## Abstract

Invasive pneumococcal disease (IPD) results from the dissemination of *Streptococcus pneumoniae* to normally sterile anatomical sites such as the bloodstream or cerebrospinal fluid. One of the primary roles of pneumococcal conjugate vaccines (PCVs) used in Romania is to reduce the burden of pneumococcal disease. This single-center retrospective study provides an updated overview of IPD epidemiology in a Romanian tertiary hospital. We analyzed 67 IPD cases identified between 2017 and 2023, of which 45 isolates underwent whole-genome sequencing followed by multilocus sequence typing. Results of genome sequence analysis revealed a diverse population of pneumococci, underlining the importance of continuous genomic surveillance. Expanded-valency pneumococcal conjugate vaccines (PCVs), particularly PCV20, showed markedly improved serotype coverage compared to PCV7 and PCV13, while PCV21 showed serotype coverage comparable to that of PCV13. Antimicrobial susceptibility testing revealed sustained resistance to beta-lactams, particularly among meningitis isolates, underscoring the need for targeted antibiotic stewardship and continuous monitoring of local resistance trends. Overall, these findings highlight the evolving epidemiology of invasive pneumococcal disease in Romania, in the post-PCV era, and the need to adapt vaccination and treatment strategies accordingly.

## 1. Introduction

*Streptococcus pneumoniae*, also known as pneumococcus, is a Gram-positive, encapsulated bacterium that is considered a normal component of the commensal flora of the upper respiratory tract. However, it is also an opportunistic pathogen, representing the most common cause of community-acquired bacterial pneumonia in both children and the elderly. Besides cases of pneumonia, *S. pneumoniae* is responsible for otitis media and invasive pneumococcal disease (IPD). IPD is characterized by the translocation of *S. pneumoniae* from its commensal niche in the upper respiratory tract to normally sterile anatomical sites, most commonly the bloodstream and cerebrospinal fluid [1,2,3]. To date, more than 100 different serotypes of *S. pneumoniae* have been described based on the structure of their polysaccharide capsule. Serotype differences have been shown to exist not only on a genetic and structural level, but also regarding the clinical manifestation of pneumococcal disease. Historically, some serotypes, such as 1, 2, 4, 5, 7F, 8, 9V, 12F, 14, 16, 18C, and 19A, have been more frequently associated with invasive disease [4,5]. It is well established that pneumococcal serotypes associated with IPD differ across geographic areas [6,7].

Despite prevention efforts, such as pneumococcal vaccination of children, IPD remains relatively prevalent both because of reduced vaccination coverage in the general population and because of serotype replacement with non-vaccine serotype pneumococci [8,9]. Thus, determining the serotype of IPD isolates is a key step for epidemiological surveillance. While historically serotyping has been done by such techniques as Quellung testing or multiplex PCR [10], whole genome sequencing (WGS) has also become a useful tool in determining pneumococcal serotype. WGS analysis of IPD isolates is also able to establish the sequence type (ST) of pneumococci via multi-locus sequence typing (MLST). MLST further distinguishes between strains, MLST enables further discrimination between strains, facilitating the identification of bacterial factors that may contribute to differences in pathogenicity [11].

The first vaccine available for use in the prevention of pneumococcal infections was a 14-valent polysaccharide vaccine, which later led to the licensing of the 23-valent polysaccharide vaccine (PPSV23) still in use today. As it offered extensive coverage against serotypes that cause 80% to 90% of IPD [4], it has been utilized successfully in disease prevention, especially in the elderly population. However, a key drawback of the PPSV23 vaccine is that it does not have sufficient immunogenic activity when used in small children [12]. Besides this limitation, it offers a limited duration of protection, which diminishes over time, and thus, revaccination is mandated in order to preserve immunity [13].

For vaccination of young children, the use of pneumococcal conjugate vaccines (PCVs) is preferred instead of PPSV23, since PCVs induce an adequate T-cell mediated immunity in this patient population when compared to non-conjugated polysaccharide vaccine [14,15]. The seven serotypes covered by the PCV7 vaccine are as follows: 4, 6B, 9V, 14, 18C, 19F, 23F. The PCV13 contains all serotypes covered by the PCV7 and extends the list with the following six serotypes: 1, 3, 5, 6A, 7F, 19A [16,17]. The PCV20 vaccine contains all the serotypes covered by PCV13, further adding seven serotypes: 8, 10A, 11A, 12F, 15B, 22F, 33F. With the addition of these 7 serotypes an extended valence conjugate vaccine is obtained, comparable to the serotype coverage of the non-conjugate PPSV23. Compared to PCV20, the coverage spectrum of PPSV23 does not include serotype 6A, meanwhile offering coverage for four additional serotypes, namely 2, 9N, 17F, and 20. The PCV21 vaccine contains only four serotypes that are covered by the PCV13 vaccine, namely 3, 6A, 7F, and 19A. In addition, the PCV21 includes serotypes 8, 9N, 10A, 11A, 12F, 15A, 15B, 16F, 17F, 20A, 22F, 23A, 23B, 24F, 31, 33F, and 35B (Table 1) [16,17]. In Romania, the conjugate vaccine formulation for vaccination of young children during our study period was PCV13, which was introduced in the National Vaccination Calendar in 2017.

Given the impact of PCV vaccines on serotype distribution and the potential emergence of non-vaccine serotypes, continued surveillance of antimicrobial susceptibility remains essential to assess their clinical and public health impact. Monitoring pneumococcal susceptibility to penicillin is a core component of the European Centre for Disease Prevention and Control (ECDC) antimicrobial resistance surveillance program. Beyond susceptibility, the term penicillin non-wild-type is used to describe isolates categorized as susceptible, increased exposure (I), or resistant (R). According to the ECDC Antimicrobial Resistance in the EU/EEA (EARS-Net) Annual Epidemiological Report for 2023, the proportion of penicillin non–wild-type *S. pneumoniae* isolates continues to rise across Europe [18]. Romania has historically performed poorly in antimicrobial resistance (AMR) surveillance and control [19], as reflected by its rate of penicillin non–wild-type invasive pneumococcal isolates, which remains above the EU average. Moreover, Romania contributed only a small number of isolates to the EARS-Net dataset, resulting in the absence of nationally representative data. This gap reflects the lack of a national surveillance program for IPD and pneumococcal antimicrobial resistance. In addition to the scarcity of centralized data, we also observed a lack of scientific publications reporting antibiotic susceptibility and serotype distribution in IPD. Most Romanian studies have focused instead on upper respiratory tract carriage. The present study, therefore, seeks to offer a local epidemiological assessment of IPD.

The objective of this study is to provide insights into the molecular epidemiology of regionally circulating strains, focusing on pneumococcal serotype distribution and possible vaccine coverage gaps.

## 2. Materials and Methods

In this single-center retrospective, descriptive study, we performed whole-genome sequencing and antimicrobial susceptibility testing on invasive pneumococcal strains isolated between 2017 and 2023 in a Romanian tertiary hospital.

### 2.1. Bacterial Strains, Patient, and Clinical Data

Invasive *S. pneumoniae* isolates obtained through routine diagnostic procedures between January 2017 and April 2023 at the Microbiology Laboratory of Mureș County Emergency Clinical Hospital were preserved at −70 °C in 20% glycerol. Duplicate isolates were defined as two or more *S. pneumoniae* isolates isolated during a single hospitalization and were excluded from the study. IPD cases were defined as *S. pneumoniae* isolated from otherwise sterile sites, such as blood or cerebrospinal fluid (CSF). Patients with IPD were categorized by age into adult and pediatric groups, the latter being defined as patients less than 18 years old. Patients were also assigned to meningitis and non-meningitis groups based on electronic chart review and site of isolation. All CSF isolates were automatically considered cases of meningitis, while patients whose charts had positive blood specimens were reviewed for clinical diagnosis or symptomatology of meningitis. The pneumococcal vaccination status of patients was not available for this study.

### 2.2. Bacterial Strains and Antimicrobial Susceptibility Testing

Antimicrobial susceptibility testing (AST) data were accessed using the centralized electronic records of the Microbiology laboratory of the Mures County Emergency Clinical Hospital. Susceptibility to antimicrobial agents was determined using disk diffusion test, gradient test, and Vitek2C (bioMérieux, Marcy l’Étoile, France) automated system, in conformity with local laboratory protocol and EUCAST (European Committee on Antimicrobial Susceptibility Testing) standard. Isolates exhibiting an oxacillin (1 µg) inhibition zone diameter ≥ 20 mm or a benzylpenicillin minimum inhibitory concentration (MIC) ≤ 0.06 mg/L were interpreted as lacking resistance mechanisms to β-lactam antibiotics. In the case of a positive oxacillin screen, further testing for benzylpenicillin, ampicillin/amoxicillin was performed either by disk diffusion or MIC determination, while ceftriaxone/cefotaxime susceptibility was assessed by determining the minimum inhibitory concentration (MIC).

### 2.3. Whole Genome Sequencing

Preserved strains were sent to the Cantacuzino National Institute for Medical-Military Research and Development for whole genome sequencing. Genomic DNA was extracted using PureLink Genomic DNA Mini kit (Invitrogen, Thermo Fisher Scientific, Waltham, MA, USA), following the manufacturer’s recommendation for Gram-positive bacteria. DNA concentrations were assessed with Qubit ds HS Assay Kit (Thermo Fisher Scientific, Waltham, MA, USA). Raw reads were generated using Illumina technology. Briefly, for library preparation Nextera XT (Illumina, San Diego, CA, USA) and IDT for Illumina DNA/RNA UD Index kit (Illumina, San Diego, CA, USA) were used, according to the kit instructions. Libraries were loaded onto NovaSeq 6000 SP Reagent Kit v1.5 (200 or 300 cycles) and sequenced on Illumina NovaSeq 6000 instrument (Illumina, San Diego, CA, USA). Raw reads were quality-filtered and de novo assembled with SKESA version 2.4.0 in SeqSphere+ software version 9.0 (Ridom GmbH, Münster, Germany). Assembled genomes were further analyzed with SeqSphere+ tools for multilocus sequence typing (*S. pneumoniae* MLST). Identification of virulence genes and antimicrobial resistance genes was achieved using web-based tools offered by the Bacterial and Viral Bioinformatics Resource Center (BV-BRC) and Proksee [20,21], utilizing a blast algorithm on the NCBI AMRFinder Plus [22] database. Sequences were deposited in the European Nucleotide Archive (ENA) under study number ERP187160.

### 2.4. Ethical Approval

Ethical approval for this study was obtained from the ethics committee of County Emergency Clinical Hospital, Târgu Mureș (reference number Ad.7875/08.04.2025).

## 3. Results

**Figure 1:**
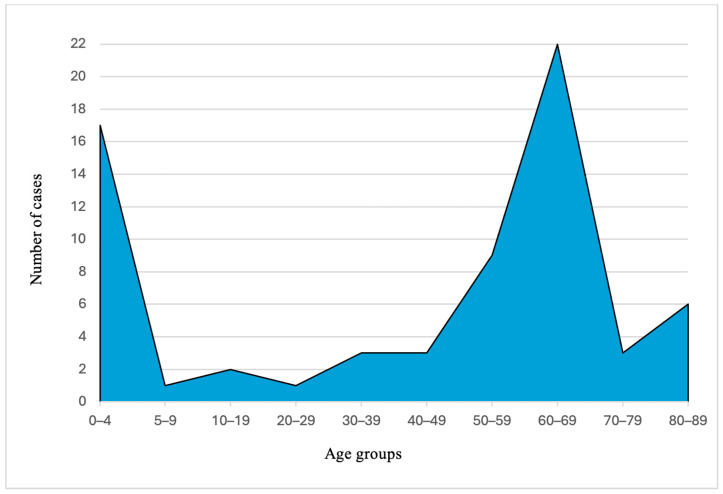
Distribution of IPD cases by age. The median age for the pediatric population was 1 year, with a range of 0 to 16 years, while the adult population presented a median age of 66, ranging from 20 to 89 years. The age distribution reveals two incidence peaks, occurring in early childhood (0–5 years) and among older adults aged 60–69 years.

**Figure 2:**
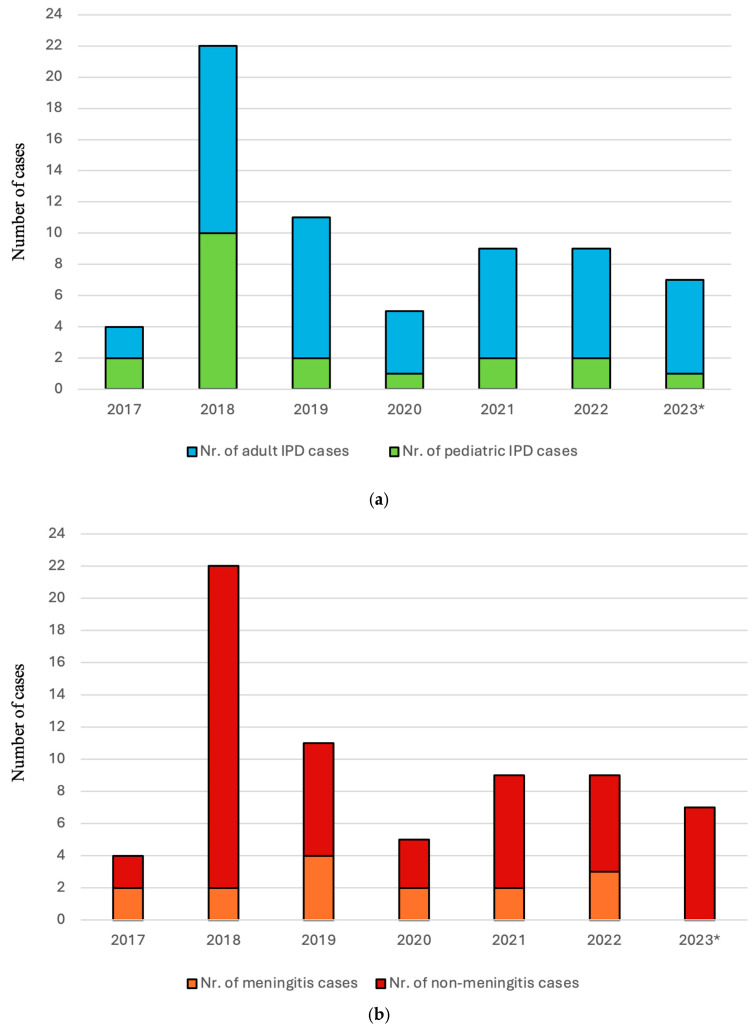
Yearly distribution of invasive *S. pneumoniae* isolates by (**a**) pediatric and adult groups and (**b**) meningitis and non-meningitis groups at Mureș County Emergency Clinical Hospital, Romania. * Not a full calendar year: cases from the year 2023 are represented only from January to April.

In total, we identified 67 cases of IPD between January 2017 and April 2023. The distribution of cases by age groups is represented in Figure 1. Yearly distribution of cases, divided into pediatric and adult groups, is represented in Figure 2a, while the distribution of meningitis and non-meningitis cases is presented in Figure 2b. In the ten cases of meningitis, samples were obtained from cerebrospinal fluid culture in seven cases and blood culture in three cases. Whole genome sequencing was performed on 47 isolates, of which two isolates were not further analyzed due to poor quality of sequencing data, resulting in 45 strains suitable for molecular description.

### 3.1. Pneumococcal Serotypes and Sequence Types Identified

**Figure 3:**
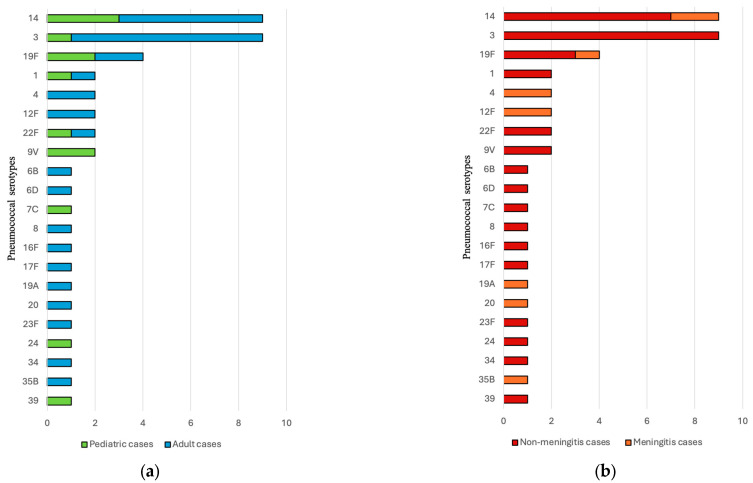
(**a**) Distribution of adult and pediatric IPD cases by pneumococcal serotype. Out of the 45 cases with pneumococcal serotype identified, we found 13 (29%) strains originating from pediatric patients. (**b**) Distribution of meningitis and non-meningitis IPD cases by pneumococcal serotype. Out of 45 cases, 22% (*n* = 10) were from cases of meningitis. In the non-meningitis group, one isolate was obtained from pleural fluid, while the other 33 strains were isolated from blood culture.

**Figure 4:**
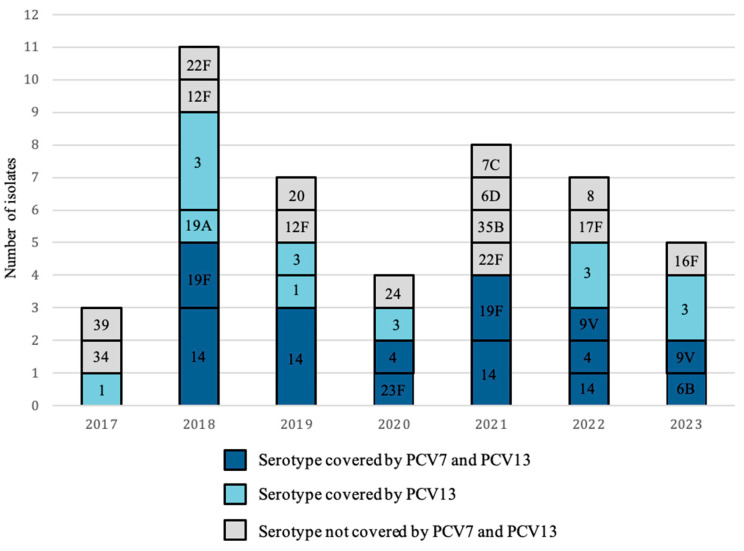
Yearly distribution of pneumococcal serotypes. Serotypes are shown inside of the bar-stacks. Colors indicate if the given serotype is covered by PCV7 or PCV13. Grey color indicates a non-PCV13 serotype. PCV13 covers all serotypes contained by PCV7.

In total, 21 different pneumococcal serotypes were identified, the most prevalent being serotype 3 and serotype 14, with 20% (*n* = 9) of isolates each, followed by serotype 19F, with a total of 8% (*n* = 4) of the cases (Figure 3a,b). The yearly distribution of serotypes is represented in Figure 4.

Multilocus Sequence Typing (MLST) of isolates resulted in the identification of 26 different sequence types (STs). We identified four novel sequence types, three of which were defined by new combinations of previously described alleles, and one by a novel allele of the *aroE* gene (Table 2).

### 3.2. Vaccine Serotypes and Vaccine Coverage

Identifying the serotype distribution of IPD isolates, we determined the vaccine serotype coverage rate and the proportion of vaccine-type isolates for five different pneumococcal vaccines, namely PCV7, PCV13, PCV20, PCV21, and PPSV23. The presumed vaccine-preventable proportion of cases was assessed based on the number of cases caused by a serotype covered by the studied vaccine (Table 3). Two IPD isolates (4%) were covered exclusively by the PCV21 formulation.

### 3.3. Antimicrobial Susceptibility

All 45 strains were initially screened for beta-lactam resistance using the 1 µg oxacillin disk diffusion method. Of these, twenty-one isolates showed a positive screening and were further tested for beta-lactam susceptibility, while 24 isolates had a negative screen, indicating beta-lactam susceptibility. Table 4 assesses the phenotypic resistance rates of the studied isolates, based on clinical manifestation. Subsequent testing showed that 58% (*n* = 26) of the isolates had susceptibility to penicillin, and a further 18% (*n* = 8) were classified as susceptible, increased exposure. Penicillin resistant strains belonged to serotypes 6B, 9V, 14, 19A, 19F, and 35B. All isolates of serotypes 14 (*n* = 9), 19F (*n* = 4), and 9V (*n* = 2) exhibited a penicillin non–wild-type phenotype. In total, 43 isolates were tested for resistance to ampicillin and amoxicillin, with 72% (*n* = 31) of the isolates showing susceptibility and a further 9% (*n* = 4) being categorized as susceptible, increased exposure, while 19% (*n* = 8) were deemed resistant to aminopenicillins.

All isolates were tested for susceptibility to third-generation cephalosporins, with 40 isolates (89%) showing susceptibility, a further 2 isolates (4%) susceptibility, increased exposure, and 3 isolates (7%) resistance to ceftriaxone and cefotaxime. Cephalosporin resistant isolates were identified in strains belonging to serotypes 14, 19A, and 19F. Out of the 11 strains showing penicillin resistance, five strains were isolated from patients with meningitis. Furthermore, isolates from meningitis were resistant to third-generation cephalosporins in 3 cases (30%). No phenotypic resistance to third-generation cephalosporins was detected among isolates from non-meningitis cases. We observed 100% susceptibility to moxifloxacin, linezolid, and vancomycin.

## 4. Discussion

Genomic analysis and surveillance have become the norm in modern epidemiological studies. As *S. pneumoniae* presents a high level of genomic variability both between serotypes and across them, sequence typing has become an essential tool to distinguish between clinical isolates of any given serotype. However, serotyping still has relevance, as vaccine protection is linked to the serotype-determining polysaccharide capsule of pneumococcus [23]. The introduction of the pneumococcal conjugate vaccines (PCV) led to a worldwide reduction of IPD cases caused by serotypes included in the PCV vaccines [24]. Because there is a lack of data on the local serotype distribution of IPD isolates in Romania, the effect of the PCV vaccines on IPD is still undetermined. The diagnostic challenges of IPD and the lack of a centralized surveillance system further reduce available data.

According to the European Centre for Disease Prevention and Control (ECDC) Annual Epidemiological Report for 2022, a total of 220 cases of IPD were reported in Romania between 2018 and 2022 [25]. Over the same period, there were 56 IPD cases identified in our study, representing a considerable proportion relative to national reports. By comparison, a total of 71,404 IPD cases were reported across 30 European Union member states during this timeframe, suggesting a notably lower reporting rate in Romania. This apparent underreporting may be caused by systemic administrative limitations as well as underdiagnosis of IPD cases. Additionally, failure to obtain adequate culture samples may further contribute to the reduced number of confirmed IPD isolates reported nationally. Analysis of the data shown in Figure 1 reveals two incidence peaks, highlighting the vulnerable populations that stand to benefit most from pneumococcal vaccination.

Regarding the yearly incidence of IPD, the data presented in Figure 2a,b indicate a drop in the incidence of IPD in 2020. Similar episodes have been identified worldwide in other community-acquired infectious diseases, such as *S. pyogenes* infections [26]. These reductions in the number of cases have been widely attributed to the COVID-19 pandemic [27]. However, we did not observe such a strong rebound effect on the incidence of IPD cases as described in the case of invasive group A streptococcal disease. A peak of disease incidence from our sample was present in 2018, with a total of 22 IPD cases identified during the calendar year. The increase may be due to multiple local interventions in 2017 focusing on blood specimen collection to improve diagnostic yield. On the other hand, a real increase in incidence cannot be ruled out. This rise could be attributed to several targeted local initiatives implemented in 2017 to enhance blood specimen collection and thereby improve diagnostic yield. However, the possibility of an actual increase in incidence cannot be excluded.

With the introduction of PCVs, pneumococcal serotype distribution has been intensively studied in both IPD and non-invasive infections. As pneumococcal vaccines target strains with a high potential for invasiveness, some studies have identified serotypes more frequently associated with IPD [5]. Furthermore, some serotypes have been identified as having a higher incidence in cases of meningitis [28,29]. In our study, serotype 3 and serotype 14 were most prevalent.

Before the introduction of pneumococcal conjugate vaccination, several studies assessing pneumococcal carriage and non-invasive disease in Romania consistently reported a predominance of classical pediatric serotypes, particularly 19F, 6B, 23F, with a lower rate of representation in the case of serotype 14 [30,31]. In nasopharyngeal carriage among children, serotypes 23F, 6B, 19F together accounted for a substantial proportion of isolates. Similarly, in non-invasive infections such as otitis media, serotypes 19A, 19F, 6A, 6B, 23F, and 14 remained dominant [32].

In contrast, our IPD data demonstrate a distinct serotype distribution, characterized by the predominance of serotype 3 and serotype 14, each accounting for 20% of invasive isolates, while serotype 19F only represented a smaller proportion (8%). This divergence underscores the well-recognized difference between carriage-associated serotypes and those with higher invasive potential, particularly serotype 3, which is infrequently detected in carriage but is disproportionately associated with invasive disease. Despite low carriage rates, serotype 3 has been associated with a high disease burden and increased mortality [30]. In the present study, serotype 3 was observed exclusively in non-meningitis cases. However, direct comparisons should be interpreted cautiously due to possible age-related differences in serotype distribution, as serotype 3 and 19A were mainly observed in adult IPD cases.

Serotype 14 is also frequently isolated from IPD cases. In our study it was present in both pediatric and adult IPD cases in relatively high proportion. The presence of serotype 14 in both pediatric carriage studies and invasive disease in our cohort highlights its dual role as both a common colonizer and an important invasive pathogen in the pre- and early vaccination era in Romania [32,33]. In recent years, multiple studies reported cases of serotype switching involving serotype 14 isolates. This phenomenon in itself can interfere with vaccine-provided protection [34,35,36,37].

Serotype 1 has been reported globally and is typically regarded as an uncommon and transient colonizer of the respiratory tract, characterized by a markedly shorter duration of carriage compared with most other pneumococcal serotypes and a high propensity for invasive disease. In line with this profile, serotype 1 was not reported in the previously discussed Romanian carriage studies, yet it was identified in our cohort as the cause of both pediatric and adult invasive pneumococcal disease, further underscoring its disproportionate association with invasive infections rather than asymptomatic colonization [38].

Serotype 4, historically linked to invasive pneumococcal disease, remains a notable concern among invasive serotypes, even though it is included in current pneumococcal vaccines [39,40,41].

Serotype prevalence is an important element used in the evaluation of vaccine coverage in cases of IPD. Even though pneumococcal conjugate vaccines have been introduced into the National Vaccination Calendar of Romania since 2017, we observed a relatively high number of pediatric IPD cases during the seven-year study period. In this context, a large proportion of IPD-associated serotypes observed in our cohort are included in currently available vaccines, such as PCV13, PCV20, and PPSV23. However, in the absence of individual vaccination status data, these observations should be interpreted as serotype coverage by available vaccines rather than evidence for lack of vaccine preventability. Furthermore, within the limitations of the sample size and study design, our data do not indicate a clear shift toward non-vaccine serotypes, although ongoing surveillance is required to reliably assess serotype replacement dynamics.

Based on global studies, vaccination of the adult population using the PPSV23 has been compared in effectiveness to the PCV13 in certain settings [42]. However, we observed a clear serotype coverage benefit from the presence of the 10 serotypes in the PPSV23 compared to PCV13. Nevertheless, the absence of vaccination status data precludes definitive conclusions regarding vaccine preventability, as infections may have occurred in unvaccinated individuals or could represent breakthrough disease in vaccinated patients. We estimated a theoretical vaccine serotype coverage under the assumption of complete vaccine efficacy, recognizing that this does not reflect real-world vaccine performance, particularly in pediatric populations where immune responses may be less robust. In our study, the presumed vaccine-preventable proportion by PPSV23 is more comparable to the rate of cases that presumably could have been prevented by PCV20. It must be emphasized that the small sample size of our study does not offer a high degree of confidence to these observations. Nonetheless, expanded-valency pneumococcal conjugate vaccines, particularly PCV20, showed markedly improved serotype coverage compared to PCV7 and PCV13, while PCV21 showed serotype coverage comparable to that of PCV13. There were also two cases identified in which the disease-causing isolates would have been covered only by the PCV21 vaccine. Based on our data, no added benefit of the PCV21 vaccine was observed compared to the PCV13 vaccine.

A high incidence rate of penicillin non-wild-type pneumococcal strains has a significant impact on empiric therapy, particularly in the case of meningitis. Although to some degree enhanced by meningeal inflammation, the penetration of beta-lactam antibiotics into the cerebrospinal fluid is considered relatively poor [43]. This pharmacokinetic limitation complicates the treatment of pneumococcal meningitis, especially when the causative agent is a penicillin non-wild-type strain. Consequently, current guidelines on empirical treatment of meningitis recommend the use of third-generation cephalosporins. In settings with high rates of penicillin non-susceptible *S. pneumoniae*, empiric inclusion of vancomycin may be appropriate [44,45]. In our study cohort, antimicrobial susceptibility testing revealed sustained resistance to beta-lactams, particularly among meningitis isolates, with more than 42% of isolates from meningitis classified as penicillin non-wild-type. Notably, there were three cases of meningitis caused by strains resistant to third-generation cephalosporins. This underscores the importance of the guideline-recommended empirical therapy, in cases of meningitis, since Romania is continually considered a country with reduced rates of penicillin susceptibility [45]. Meanwhile improving diagnostic efforts should present a high priority, to facilitate susceptibility testing and consequent deescalation of antimicrobial therapy.

Besides the marked beta-lactam resistance, we also observed a high rate of resistance to erythromycin. Pneumococcal resistance to macrolides has been increasing worldwide, thus removing valuable therapeutic options in case of beta-lactam-resistant infections [46].

These findings are consistent with earlier Romanian data on non-invasive pneumococcal infections, which also reported very high rates of penicillin non-susceptibility. In a 2017 study investigating pneumococcal isolates from acute otitis media (AOM) and community-acquired alveolar pneumonia (CAAP), more than 90% of isolates in both clinical contexts were non-susceptible to penicillin, with a substantial proportion exhibiting high-level resistance [32]. These high rates of antibiotic resistance are alarming, considering IPD is most commonly a community-acquired infection. This suggests the existence of a shared, resistant pneumococcal reservoir circulating in the community and serving as a potential source for severe invasive infections. A sustained susceptibility toward vancomycin, linezolid, and moxifloxacin constitutes the last therapeutic options against drug-resistant pneumococcal infections. Thus, our findings help underscore the need for targeted antibiotic stewardship and continuous monitoring of local resistance trends.

The principal limitation of our study is its single-center design, which may limit the generalizability of our conclusions. Despite its long duration, the number of cases included is relatively small. However, these cases constitute a great proportion of the IPD cases identified during the study period. This suggests that, in the absence of microbiological diagnosis, IPD cases are overlooked. Thus, a further limitation is that the retrospective nature of the study limited our ability to influence sample collection, potentially restricting case identification. A further limitation was the unavailability of patient vaccination status data. However, we must emphasize that, to our knowledge, this is the first study to analyze the antimicrobial resistance profile and use whole genome sequencing to determine serotype and sequence type distribution of IPD isolates in Romania.

## 5. Conclusions

Reliable data on IPD remain limited in Romania. Given that the country ranks among the most vulnerable EU states regarding multidrug-resistant microorganisms, community-associated infections with *S. pneumoniae* pose a significant threat. Thus, national-level IPD surveillance and integration with larger studies are paramount. Achieving this objective depends on improved diagnostic capabilities and more consistent sample collection habits. Our results of genome sequence analysis reveal a diverse population of pneumococci, underlining the importance of continuous genomic surveillance. The overwhelming presence of PCV7 and PCV13 vaccine types in our sample indicates a lack of vaccination in both the pediatric and adult populations. While phenotypic characterization, such as the antimicrobial susceptibility pattern is a useful epidemiological tool, whole-genome sequencing of pneumococcal isolates is informative in understanding pneumococcal strain distribution and assessing local trends in IPD. Vaccination of all age groups is a highly effective tool in combating IPD.

## Figures and Tables

**Table 1:** Vaccine coverage of the different pneumococcal vaccines. Grey area indicates vaccine coverage of the serotype indicated in the left column. PCV—pneumococcal conjugate vaccine; PPSV—pneumococcal polysaccharide vaccine.

	PCV7	PCV13	PCV20	PCV21	PPSV23
1					
2					
3					
4					
5					
6A					
6B					
7F					
8					
9N					
9V					
10A					
11A					
12F					
14					
15A					
15B					
16F					
17F					
18C					
19A					
19F					
20/20A					
22F					
23A					
23B					
23F					
24F					
31					
33F					
35B					

**Table 2:** Number of identified pneumococcal sequence types and serotypes. The second column of the table indicates which of the vaccines included in our study offer coverage for the given serotype.

Serotype	Vaccine Coverage	Sequence Type	Number of Isolates
1	PCV13, PCV20, PPSV23	306	2
3	PCV13, PCV20, PCV21, PPSV23	180	3
		260	1
		505	3
		1377	2
4	PCV7, PCV13, PCV20, PPSV23	205	2
6B	PCV7, PCV13, PCV20, PPSV23	1143	1
6D	Non-vaccine serotype	20,514	1
7C	Non-vaccine serotype	20,591	1
8	PCV20, PCV21, PPSV23	1480	1
9V	PCV7, PCV13, PCV20, PPSV23	156	1
		166	1
12F	PCV20, PCV21, PPSV23	8060	1
		15,340	1
14	PCV7, PCV13, PCV20, PPSV23	15	3
		44	3
		2944	3
16F	PCV21	14,663	1
17F	PCV21, PPSV23	20,360	1
19A	PCV13, PCV20, PCV21, PPSV23	3772	1
19F	PCV7, PCV13, PCV20, PPSV23	179	1
		271	1
		423	2
20	PCV21, PPSV23	1026	1
22F	PCV20, PCV21, PPSV23	433	2
23F	PCV7, PCV13, PCV20, PPSV23	20,513	1
24	PCV21	4253	1
34	Non-vaccine serotype	4083	1
35B	PCV21	558	1

**Table 3:** Presumed vaccine-preventable proportion of cases for the different pneumococcal vaccines. In this study, the term presumed vaccine-preventable portion is defined as the number of IPD cases attributable to isolates belonging to serotypes targeted by a given vaccine formulation.

Vaccine Name	Presumed Vaccine-Preventable Proportion of Cases				
	Total Cases *n* (%)	Meningitis *n* (%)	Non-Meningitis *n* (%)	Adult *n* (%)	Pediatric *n* (%)
PCV7	19 (49)	5 (50)	14 (40)	12 (54)	7 (38)
PCV13	31 (69)	6 (60)	25 (71)	22 (69)	9 (69)
PCV20	36 (80)	8 (80)	28 (80)	26 (81)	10 (77)
PCV21	20 (44)	5 (50)	15 (43)	18 (56)	2 (15)
PPSV23	38 (84)	9 (90)	29 (83)	28 (88)	10 (77)

**Table 4:** Antimicrobial resistance profiles of invasive *S. pneumoniae* isolates by age group and meningitis and non-meningitis etiology. Antimicrobial susceptibility testing was interpreted in conformity with EUCAST standard. S-susceptible; I-susceptible, increased exposure; R-resistant.

Antimicrobial Agent	Phenotype	Total Rate *n* (%)	Adult Cases *n* (%)	Pediatric Cases *n* (%)	Meningitis *n* (%)	Non-Meningitis *n* (%)
Penicillin	S	26 (58)	21 (66)	5 (38)	5 (50)	21 (60)
	I	8 (18)	3 (9)	5 (38)	0 (0)	8 (23)
	R	11 (24)	8 (25)	3 (23)	5 (50)	6 (17)
Aminopenicillins	S	31 (72)	23 (72)	8 (73)	6 (60)	25 (76)
	I	4 (9)	4 (12)	0 (0)	2 (20)	2 (6)
	R	8 (19)	5 (16)	3 (27)	2 (20)	6 (18)
Third-generation cephalosporins	S	40 (89)	27 (84)	13 (100)	7 (70)	33 (94)
	I	2 (4)	2 (6)	0 (0)	0 (0)	2 (6)
	R	3 (7)	3 (10)	0 (0)	3 (30)	0 (0)
Erythromycin	S	30 (66)	23 (72)	7 (54)	7 (70)	23 (66)
	R	15 (33)	9 (28)	6 (46)	3 (30)	12 (34)
Trimethoprim/ sulfamethoxazole	S	30 (68)	22 (69)	8 (67)	6 (60)	24 (83)
	R	14 (32)	10 (31)	4 (33)	4 (40)	10 (18)
Clindamycin	S	31 (74)	25 (81)	6 (55)	8 (80)	23 (72)
	R	11 (26)	6 (19)	5 (45)	2 (20)	9 (28)
Tetracycline	S	35 (80)	25 (81)	10 (77)	6 (67)	29 (83)
	R	9 (20)	6 (19)	3 (23)	3 (33)	6 (17)

## Data Availability

All used genome sequences have been uploaded into the European Nucleotide Archive (ENA) under study number ERP187160. Further data presented in this study are available on request from the corresponding author.

## Abbreviations

The following abbreviations are used in this manuscript:

IPD	Invasive pneumococcal disease
WGS	Whole genome sequencing
PCV	Pneumococcal conjugate vaccine
PPSV	Pneumococcal polysaccharide vaccine

## References

[1] Henriques-Normark B., Tuomanen E.I. (2013). The pneumococcus: Epidemiology, microbiology, and pathogenesis. Cold Spring Harb. Perspect. Med..

[2] Lynch J.P., Zhanel G.G. (2009). Streptococcus pneumoniae: Epidemiology, risk factors, and strategies for prevention. Semin. Respir. Crit. Care Med..

[3] Weiser J.N., Ferreira D.M., Paton J.C. (2018). Streptococcus pneumoniae: Transmission, colonization and invasion. Nat. Rev. Microbiol..

[4] Scelfo C., Menzella F., Fontana M., Ghidoni G., Galeone C., Facciolongo N.C. (2021). Pneumonia and Invasive Pneumococcal Diseases: The Role of Pneumococcal Conjugate Vaccine in the Era of Multi-Drug Resistance. Vaccines.

[5] Song J.Y., Nahm M.H., Moseley M.A. (2013). Clinical Implications of Pneumococcal Serotypes: Invasive Disease Potential, Clinical Presentations, and Antibiotic Resistance. J. Korean Med. Sci..

[6] Quesada M.G., Peterson E.M., Bennett J.C., Hayford K., Zeger S.L., Yang Y., Hetrich M.K., Feikin D.R., Cohen A.L., von Gottberg A. (2025). Serotype distribution of remaining invasive pneumococcal disease after extensive use of ten-valent and 13-valent pneumococcal conjugate vaccines (the PSERENADE project): A global surveillance analysis. Lancet Infect. Dis..

[7] Maeda H., Morimoto K. (2025). Global distribution and characteristics of pneumococcal serotypes in adults. Hum. Vaccin. Immunother..

[8] Diab-Casares L., Tormo-Palop N., Hernández-Felices F.J., Artal-Muñoz V., Floría-Baquero P., Martin-Rodríguez J.L., Medina-González R., Cortés-Badenes S., Fuster-Escrivá B., Gil-Bruixola A. (2025). Predominant Pneumococcal Serotypes in Isolates Causing Invasive Disease in a Spanish Region: An Examination of Their Association with Clinical Factors, Antimicrobial Resistance, and Vaccination Coverage. J. Clin. Med..

[9] Wantuch P.L., Avci F.Y. (2018). Current status and future directions of invasive pneumococcal diseases and prophylactic approaches to control them. Hum. Vaccin. Immunother..

[10] Siira L., Kaijalainen T., Lambertsen L., Nahm M.H., Toropainen M., Virolainena A. (2012). From Quellung to Multiplex PCR, and Back When Needed, in Pneumococcal Serotyping. J. Clin. Microbiol..

[11] D’aEth J.C., Bertran M., Abdullahi F., Eletu S., Hani E., Fry N.K., Ladhani S.N., Litt D.J. (2025). Whole-genome sequencing, strain composition, and predicted antimicrobial resistance of Streptococcus pneumoniae causing invasive disease in England in 2017-20: A prospective national surveillance study. Lancet Microbe.

[12] Daniels C.C., Rogers P.D., Shelton C.M. (2016). A Review of Pneumococcal Vaccines: Current Polysaccharide Vaccine Recommendations and Future Protein Antigens. J. Pediatr. Pharmacol. Ther. JPPT.

[13] Grabenstein J.D., Manoff S.B. (2012). Pneumococcal polysaccharide 23-valent vaccine: Long-term persistence of circulating antibody and immunogenicity and safety after revaccination in adults. Vaccine.

[14] Thong B.Y.H., Pawankar R., Park H.S., Latiff A.H.A. (2023). Evaluating immune responses to pneumococcal vaccines. Asia Pac. Assoc. Allergy Asthma Clin. Immunol..

[15] Pneumococcal Disease|The Australian Immunisation Handbook. https://immunisationhandbook.health.gov.au/contents/vaccine-preventable-diseases/pneumococcal-disease.

[16] About Pneumococcal Vaccines: For Providers|CDC. https://www.cdc.gov/vaccines/vpd/pneumo/hcp/about-vaccine.html.

[17] Greene C.M., Kyaw M.H., Ray S.M., Schaffner W., Lynfield R., Barrett N.L., Long C., Gershman K., Pilishvili T., Roberson A. (2006). Preventability of invasive pneumococcal disease and assessment of current polysaccharide vaccine recommendations for adults: United States, 2001–2003. Clin. Infect. Dis..

[18] European Centre for Disease Prevention and Control (ECDC) (2024). Antimicrobial Resistance in the EU/EEA (EARS-Net) Annual Epidemiological Report for 2023.

[19] Kohlenberg A., Monnet D.L., Nilsson A., Borg M., Shetty N. (2017). ECDC Country Visit to Romania to Discuss Antimicrobial Issues: 6–10 March 2017.

[20] Grant J.R., Enns E., Marinier E., Mandal A., Herman E.K., Chen C.-Y., Graham M., Van Domselaar G., Stothard P. (2023). Proksee: In-depth characterization and visualization of bacterial genomes. Nucleic Acids Res..

[21] Olson R.D., Assaf R., Brettin T., Conrad N., Cucinell C., Davis J.J., Dempsey D.M., Dickerman A., Dietrich E.M., Kenyon R.W. (2023). Introducing the Bacterial and Viral Bioinformatics Resource Center (BV-BRC): A resource combining PATRIC, IRD and ViPR. Nucleic Acids Res..

[22] Feldgarden M., Brover V., Gonzalez-Escalona N., Frye J.G., Haendiges J., Haft D.H., Hoffmann M., Pettengill J.B., Prasad A.B., Tillman G.E. (2021). AMRFinderPlus and the Reference Gene Catalog facilitate examination of the genomic links among antimicrobial resistance, stress response, and virulence. Sci. Rep..

[23] Weinberger D.M., Trzciński K., Lu Y.-J., Bogaert D., Brandes A., Galagan J., Anderson P.W., Malley R., Lipsitch M. (2009). Pneumococcal capsular polysaccharide structure predicts serotype prevalence. PLoS Pathog..

[24] Falup-Pecurariu O. (2012). Lessons Learnt after the Introduction of the Seven Valent-Pneumococcal Conjugate Vaccine Toward Broader Spectrum Conjugate Vaccines. Biomed. J..

[25] European Centre for Disease Prevention and Control (ECDC) (2024). Invasive Pneumococcal Disease–Annual Epidemiological Report for 2022.

[26] Nygaard U., Hartling U.B., Munkstrup C., Nielsen A.B., Dungu K.H.S., Schmidt L.S., Glenthøj J., Matthesen A.T., Rytter M.J.H., Holm M. (2024). Invasive group A streptococcal infections in children and adolescents in Denmark during 2022–23 compared with 2016–17 to 2021–22: A nationwide, multicentre, population-based cohort study. Lancet Child Adolesc. Health.

[27] Goldberg-Bockhorn E., Hagemann B., Furitsch M., Hoffmann T.K. (2024). Invasive Group A Streptococcal Infections in Europe After the COVID-19 Pandemic. Dtsch. Arztebl. Int..

[28] Gessner B.D., Sanou O., Drabo A., Tamekloe T.A., Yaro S., Tall H., Moïsi J.C., Mueller J.E., Njanpop-LaFourcade B.-M. (2012). Pneumococcal serotype distribution among meningitis cases from Togo and Burkina Faso during 2007–2009. Vaccine.

[29] Sanz J.C., de Miguel S., Ordobás M., Comas L.G. (2020). Streptococcus pneumoniae serotypes with meningeal tropism in cases of invasive pneumococcal disease. Community of Madrid, 2007–2018. Enferm. Infecc. Microbiol. Clin..

[30] Falup-Pecurariu O., Leibovitz E., Mercas A., Bleotu L., Zavarache C., Porat N., Dagan R., Greenberg D. (2013). Pneumococcal acute otitis media in infants and children in central Romania, 2009–2011: Microbiological characteristics and potential coverage by pneumococcal conjugate vaccines. Int. J. Infect. Dis..

[31] Luminos M., Dorobat O., Jugulete G., Popescu G.A., Florea D., Draganescu A., Cercel A.S., Rafila A. (2014). Nasopharyngeal carriage of Streptococcus pneumoniae in Romanian children before the introduction of the pneumococcal conjugated vaccination into the national immunization programme: A national, multi-centre, cross-sectional observational study. Int. J. Infect. Dis..

[32] Lixandru R.-I., Falup-Pecurariu C., Bleotu L., Mercas A., Leibovitz E., Dagan R., Greenberg D., Falup-Pecurariu O. (2017). Streptococcus pneumoniae serotypes and antibiotic susceptibility patterns in middle ear fluid isolates during acute otitis media and nasopharyngeal isolates during community-acquired alveolar pneumonia in central Romania. Pediatr. Infect. Dis. J..

[33] Falup-Pecurariu O., Bleotu L., Zavarache C., Peled N.M., Anton O., Robu M., Falup-Pecurariu C., Rogozea L., Porat N., Greenberg D. (2011). Streptococcus pneumoniae nasopharyngeal colonization in children in brasov, central romania: High antibiotic resistance and coverage by conjugate vaccines. Pediatr. Infect. Dis. J..

[34] Darkwah S., Somda N.S., Mahazu S., Donkor E.S. (2025). Pneumococcal serotypes and their association with death risk in invasive pneumococcal disease: A systematic review and meta-analysis. Front. Med. SA.

[35] Manna S., Spry L., Wee-Hee A., Ortika B.D., Boelsen L.K., Batinovic S., Mazarakis N., Ford R.L., Lo S.W., Bentley S.D. (2022). Variants of Streptococcus pneumoniae Serotype 14 from Papua New Guinea with the Potential to Be Mistyped and Escape Vaccine-Induced Protection. Microbiol. Spectr..

[36] Yue J., Chen L., Yao T., Du P., Gu C., Wei H., Han K., Rong C., Wang C., Zhang Q. (2025). A global epidemic serotype 14 Streptococcus pneumoniae switching to non-vaccine types. Microbiol. Spectr..

[37] Zhou M., Wang Z., Wang Z., Zhang L., Zhang L., Kudinha T., Kudinha T., An H., An H., Qian C. (2022). Serotype Distribution, Antimicrobial Susceptibility, Multilocus Sequencing Type and Virulence of Invasive Streptococcus pneumoniae in China: A Six-Year Multicenter Study. Front. Microbiol..

[38] Chaguza C., Yang M., Jacques L.C., Bentley S.D., Kadioglu A. (2021). Serotype 1 pneumococcus: Epidemiology, genomics, and disease mechanisms. Trends Microbiol..

[39] Beall B., Walker H., Tran T., Li Z., Varghese J., McGee L., Li Y., Metcalf B.J., Gierke R., Mosites E. (2021). Upsurge of Conjugate Vaccine Serotype 4 Invasive Pneumococcal Disease Clusters Among Adults Experiencing Homelessness in California, Colorado, and New Mexico. J. Infect. Dis..

[40] Center for Indigenous Health (2024). ST4 Alert for NN Providers_2024.0312. Active Bacterial Surveillance Alert.

[41] Centers for Disease Control and Prevention Summary of Risk-Based Pneumococcal Vaccination Recommendations/Pneumococcal/CDC. https://www.cdc.gov/pneumococcal/hcp/vaccine-recommendations/risk-indications.html.

[42] World Health Organization (WHO) (2021). Considerations for pneumococcal vaccination in older adults. Wkly. Epidemiol. Rec..

[43] Nau R., Sörgel F., Eiffert H. (2010). Penetration of Drugs through the Blood-Cerebrospinal Fluid/Blood-Brain Barrier for Treatment of Central Nervous System Infections. Clin. Microbiol. Rev..

[44] World Health Organization (2025). WHO Guidelines on Meningitis Diagnosis, Treatment and Care.

[45] van de Beek D., Cabellos C., Dzupova O., Esposito S., Klein M., Kloek A., Leib S., Mourvillier B., Ostergaard C., Pagliano P. (2016). ESCMID guideline: Diagnosis and treatment of acute bacterial meningitis. Clin. Microbiol. Infect..

[46] Gonzales B.E., Mercado E.H., Pinedo-Bardales M., Hinostroza N., Campos F., Chaparro E., Del Águila O., Castillo M.E., Saenz A., Reyes I. (2022). Increase of Macrolide-Resistance in Streptococcus pneumoniae Strains After the Introduction of the 13-Valent Pneumococcal Conjugate Vaccine in Lima, Peru. Front. Cell. Infect. Microbiol..

